# Supplementary material for: MetaRibo-Seq measures translation in microbiomes
Source: Nat Commun. 2020 Jun 29;11:3268. doi: 10.1038/s41467-020-17081-z (PMC7324362; doi:10.1038/s41467-020-17081-z)
Supplement: Supplementary file 10 — Supplementary Data 7 [file 41467_2020_17081_MOESM10_ESM.zip › File2/Confidence_High_Taxonomy/57742_out.krona.html]

Javascript must be enabled to view this page.

members
magnitude
magnitudeUnassigned
count
unassigned
taxon
rank

57742\_out

44

2
superkingdom
44

1239
phylum
44

44
186801
class

44
186802
order

44
family
31979

44
1485
genus

59620
species

SRS011239\_contig\_number\_25782SRS013476\_contig\_number\_36620SRS013951\_contig\_number\_33835SRS014459\_contig\_number\_8815SRS015065\_contig\_number\_36678SRS015794\_contig\_number\_2924SRS016018\_contig\_number\_12677SRS018817\_contig\_number\_contig-100\_4874.80384SRS018836\_contig\_number\_25003SRS019496\_contig\_number\_678SRS020233\_contig\_number\_59730SRS020869\_contig\_number\_contig-100\_1295.226245SRS024132\_contig\_number\_21494SRS024331\_contig\_number\_35609SRS024435\_contig\_number\_31653SRS045645\_contig\_number\_15785SRS049896\_contig\_number\_contig-100\_1117.194680SRS053356\_contig\_number\_32081SRS053398\_contig\_number\_19904SRS053573\_contig\_number\_2887SRS054905\_contig\_number\_10727SRS063040\_contig\_number\_45425SRS076756\_contig\_number\_21695SRS076804\_contig\_number\_20741SRS076976\_contig\_number\_21381SRS077024\_contig\_number\_13044SRS078419\_contig\_number\_34886SRS098881\_contig\_number\_24666SRS103987\_contig\_number\_31827SRS1041133\_contig\_number\_19521SRS1041134\_contig\_number\_3627SRS142923\_contig\_number\_19553SRS143895\_contig\_number\_contig-100\_2122.265529SRS144506\_contig\_number\_6363SRS147139\_contig\_number\_33556SRS147614\_contig\_number\_contig-100\_1223.69434SRS147977\_contig\_number\_contig-100\_1224.88593SRS148319\_contig\_number\_17348SRS148424\_contig\_number\_29517SRS149181\_contig\_number\_26614SRS149784\_contig\_number\_24228SRS893270\_contig\_number\_8028SRS893342\_contig\_number\_4500SRS971276\_contig\_number\_10800
44
